# Genetic Algorithms for Optimized Diagnosis of Alzheimer’s Disease and Frontotemporal Dementia Using Fluorodeoxyglucose Positron Emission Tomography Imaging

**DOI:** 10.3389/fnagi.2021.708932

**Published:** 2022-02-03

**Authors:** Josefa Díaz-Álvarez, Jordi A. Matias-Guiu, María Nieves Cabrera-Martín, Vanesa Pytel, Ignacio Segovia-Ríos, Fernando García-Gutiérrez, Laura Hernández-Lorenzo, Jorge Matias-Guiu, José Luis Carreras, José L. Ayala, Alzheimer’s Disease Neuroimaging Initiative

**Affiliations:** Alzheimer’s Association; Alzheimer’s Drug Discovery Foundation; Araclon Biotech; BioClinica, Inc.; Biogen; Bristol-Myers Squibb Company; CereSpir, Inc.; Cogstate; Eisai Inc.; Elan Pharmaceuticals, Inc.; Eli Lilly and Company; EuroImmun; F. Hoffmann-La Roche Ltd. and its affiliated company Genentech, Inc.; Fujirebio; GE Healthcare; IXICO Ltd.; Janssen Alzheimer Immunotherapy Research Development, LLC.; Johnson Johnson Pharmaceutical Research Development LLC.; Lumosity; Lundbeck; Merck Co., Inc.; Meso Scale Diagnostics, LLC.; NeuroRx Research; Neurotrack Technologies; Novartis Pharmaceuticals Corporation; Pfizer Inc.; Piramal Imaging; Servier; Takeda Pharmaceutical Company; and Transition Therapeutics; ^1^Department of Computer Architecture and Communications, Centro Universitario de Mérida, Universidad de Extremadura, Badajoz, Spain; ^2^Department of Neurology, Hospital Clinico San Carlos, San Carlos Research Health Institute (IdISSC), Universidad Complutense, Madrid, Spain; ^3^Department of Nuclear Medicine, Hospital Clinico San Carlos, San Carlos Research Health Institute (IdISSC), Universidad Complutense, Madrid, Spain; ^4^Department of Computer Architecture and Automation, Universidad Complutense, Madrid, Spain

**Keywords:** positron emission tomography, Alzheimer’s disease, frontotemporal dementia, primary progressive aphasia, machine learning, unsupervised algorithm, genetic algorithm, evolutionary algorithm

## Abstract

Genetic algorithms have a proven capability to explore a large space of solutions, and deal with very large numbers of input features. We hypothesized that the application of these algorithms to ^18^F-Fluorodeoxyglucose Positron Emission Tomography (FDG-PET) may help in diagnosis of Alzheimer’s disease (AD) and Frontotemporal Dementia (FTD) by selecting the most meaningful features and automating diagnosis. We aimed to develop algorithms for the three main issues in the diagnosis: discrimination between patients with AD or FTD and healthy controls (HC), differential diagnosis between behavioral FTD (bvFTD) and AD, and differential diagnosis between primary progressive aphasia (PPA) variants. Genetic algorithms, customized with *K-Nearest Neighbor* and *BayesNet Naives* as the fitness function, were developed and compared with Principal Component Analysis (PCA). K-fold cross validation within the same sample and external validation with ADNI-3 samples were performed. External validation was performed for the algorithms distinguishing AD and HC. Our study supports the use of FDG-PET imaging, which allowed a very high accuracy rate for the diagnosis of AD, FTD, and related disorders. Genetic algorithms identified the most meaningful features with the minimum set of features, which may be relevant for automated assessment of brain FDG-PET images. Overall, our study contributes to the development of an automated, and optimized diagnosis of neurodegenerative disorders using brain metabolism.

## Introduction

Alzheimer’s disease (AD) and Frontotemporal dementia (FTD) are among the most frequent neurodegenerative disorders causing cognitive impairment and dementia (Bang et al., 2015). Clinical diagnosis is often challenging, because early differential diagnosis may be difficult. AD usually presents with memory loss, which is a frequent symptom in the general population. FTD may present with behavioral changes, executive dysfunction, or language disorders (Fernández-Matarrubia et al., 2014). According to the clinical presentation of FTD, three core disorders are recognized: the behavioral variant FTD (bvFTD), non-fluent primary progressive aphasia (nfPPA), and semantic variant primary progressive aphasia (svPPA). Diagnostic work-up involves several procedures including neurological examination, neuropsychological assessment, neuroimaging techniques [preferably magnetic resonance imaging (MRI) and/or positron emission tomography (PET)], CSF biomarkers, and genetics techniques.

In the setting of a potential AD or FTD diagnosis, three main clinical questions arise. First, do memory loss or behavioral symptoms signal the onset of a neurodegenerative disorder? Memory or behavioral symptoms in early stages may be non-specific, and may be normal, or explained by non-neurodegenerative causes such as vascular damage, personality changes or psychiatric disorders, among others (Devenney et al., 2018). Second, is the cause AD or FTD? Behavioral alterations and memory loss may present in both AD and FTD; thus, differential diagnosis between these entities may be challenging. Third, in cases with language impairment presentation, what variant of PPA does the patient have? In patients with word-finding difficulties, differential diagnosis between the three main variants of PPA (nfPPA, svPPA, and logopenic PPA) may be difficult (Marshall et al., 2018), and accurate classification is important considering the different underlying pathology, treatment, and outcomes for each subtype (Matias-Guiu et al., 2015a).

Positron Emission Tomography (PET) technology has progressed considerably, leading to new methods for early and differential diagnosis of dementia (Zukotynski et al., 2018). Although several tracers have been developed and studied, ^18^F-fluorodeoxyglucose (FDG) is probably the most used and available. FDG-PET represents a unique, minimally invasive tool for the evaluation of brain metabolism. However, FDG-PET requires interpretation by neuroimaging specialists with a high level of training (Matias-Guiu et al., 2015b). In addition, there is currently a general lack of evidence to strongly recommend the routine use of FDG-PET for the diagnosis of dementia (Bouwman et al., 2018; Nestor et al., 2018).

Machine learning may assist clinical diagnostic decisions by automatically classifying and predicting dementia using computer-aided diagnosis techniques. There is broad recognition that machine learning may assist in addressing the increasing complexity and volume of imaging data. However, it is in the acquisition of knowledge from multiple heterogeneous data sources that machine learning confers the greatest advantage. Importantly, these techniques are able not only to automate diagnosis, but also to select the most relevant features. This may be very relevant in simplifying the diagnostic process of some disorders, such as FTD and its variants, in which a wide range of techniques and a high level of expertise are necessary to achieve an accurate diagnosis. The contributions of machine learning to the field of dementia have recently been reviewed (Habes et al., 2020). Previously, traditional regression modeling techniques had been applied to clinical data to identify early cases of AD and related dementias (So et al., 2017), to cluster patients into fast vs. slow progression sub-types (Gamberger et al., 2017), to distinguish mild cognitive impairment or normal ageing from early dementia (Shankle et al., 1997), and to assist in the interpretation and clinical significance of findings from neuroimaging studies (Callahan et al., 1995; Lao et al., 2004; Li et al., 2007; Klippel et al., 2008; Dyrba et al., 2015). Very recent approaches (Nori et al., 2019) have also aimed to build machine learning models to predict incident mild cognitive impairment, AD, and related dementias using structured data from administrative sources and electronic health records. In multivariate classification, and to enable early diagnosis of dementia, Support Vector Machine is the most frequently used classifier (Nanni et al., 2018). In the setting of FTD, machine learning algorithms have been applied very recently in some studies, generally with relatively small sample sizes. In this regard, they have been used to improve differential diagnosis between FTD and AD using multimodal MRI (Kim et al., 2019) or combining cognitive tests and MRI (Bachli et al., 2020), to discriminate between patients with FTD and healthy controls (HC) using structural MRI (Donnelly-Kehoe et al., 2019), and in the early diagnosis of presymptomatic mutation carriers using MRI (Feis et al., 2019). In addition, clustering techniques have been used to capture different clinical and/or neuroimaging patterns of both bvFTD and PPA, due to the heterogeneity of these disorders (Whitwell et al., 2009; Matias-Guiu et al., 2019).

In this study, we hypothesized that machine learning algorithms, and specifically genetic algorithms, may help in the diagnosis of AD and FTD by selecting the most meaningful features in FDG-PET images and automating diagnosis. We aimed to apply machine learning techniques for the development of computer-aided diagnosis models. We developed a genetic algorithm-based methodology capable of minimizing the required input data for FDG-PET imaging, in order to achieve the highest accuracy with the minimum set of data. Specifically, algorithms were developed for the three main clinical questions previously outlined in the setting of AD and FTD: first, detection of AD and FTD in comparison to a healthy control group; second, differential diagnosis between AD and bvFTD; and third, differential diagnosis between PPA variants. To this end, we fed our algorithms with data from a large cohort of well-characterized patients with AD, bvFTD, and PPA.

## Methodology

### Study Population

Patients were prospectively recruited from the Department of Neurology of our hospital. All patients were evaluated using a comprehensive neuropsychological protocol and FDG-PET imaging. Only patients with at least 2 years of follow-up confirming the diagnosis were enrolled in this study. The following diagnostic groups were included: (a) Patients with bvFTD (*n* = 81); (b) Patients with PPA, categorized into the three main clinical variants (non-fluent, semantic, and logopenic) (*n* = 68); (c) Patients with Alzheimer’s disease (*n* = 88); and d) Healthy controls (HC) (*n* = 39).

All patients met the current diagnostic criteria (Gorno-Tempini et al., 2011; McKhann et al., 2011; Rascovsky et al., 2011). At the moment of FDG-PET imaging, patients were at mild or very mild stages of dementia according to the Clinical Dementia Rating (Morris, 1993). The main characteristics of the sample are shown in Table 1. All patients were recruited from our center and underwent a common diagnostic protocol. All patients meeting the inclusion criteria and examined between July 2014 and December 2018 were included.

**TABLE 1:** Main demographic characteristics.

	**AD (*n* = 88)**	**bvFTD (*n* = 81)**	**PPA (*n* = 68)**	**HC(*n* = 39)**
Age (year)	73.90 ± 9.51	70.68 ± 8.36	72.62 ± 8.00	68.06 ± 5.67
Women n (%)	47 (53.4%)	36 (44.4%)	39 (57.4%)	24 (75.0%)
Years of education	9.51 ± 4.58	9.40 ± 4.69	11.84 ± 4.85	12.21 ± 4.70
ACE-III	68.06 ± 16.25	60.06 ± 21.06	60.44 ± 17.89	89.87 ± 5.79
MMSE	24.06 ± 4.27	21.91 ± 6.75	23.90 ± 5.26	29.15 ± 1.27

All subjects and/or their legal representatives gave written informed consent to participate in the study, which was approved by the local research ethics committee from the Hospital Clinico San Carlos. Research was performed in accordance with the Declaration of Helsinki and its amendments.

According to the aims of the study, the sample was divided into the following datasets:

(a) 81 patients with bvFTD and 39 HCs; (b) 88 patients with AD and 81 with bvFTD; (c) 88 patients with AD and 39 HCs; and (d) 68 patients with PPA and 17 healthy controls.

### Clinical and Cognitive Assessments

All patients were evaluated with a comprehensive neuropsychological protocol, including a standardized neuropsychological battery that has been normalized and validated in our setting (Peña-Casanova et al., 2009), and several language tests developed by our group. The general cognitive examination included the following tests: Addenbrooke’s Cognitive Examination III, Corsi block-tapping test, Trail Making Test, Symbol Digit Modalities Test, Stroop Color-Word Interference Test, Free and Cued Selective Reminding Test, Rey-Osterrieth Complex Figure (copy and recall), Visual Object and Space Perception Battery, and Tower of London. Language protocol was administered to patients with PPA and it consisted of the following tasks elaborated by our group: picture naming, action naming, word-picture matching, action-verb matching, synonym judgment, semantic association, initial phoneme deletion, word spelling, non-word repetition, forward and backward digit span, reading (words, foreign words, words without stress marks, and non-words), verbal repetition (syllables, pairs of syllables, words, pairs of words, non-words, and sentences), complex sentence comprehension, constrained verb production, buccofacial praxis, and verbal fluency (animals, words beginning with “p,” and actions). In addition, spontaneous speech was evaluated with the “Cookie Theft” from the Boston Diagnostic Aphasia Examination. Further details about neuropsychological assessments are specified elsewhere (Fernández-Matarrubia et al., 2017; Matias-Guiu et al., 2017, 2018, 2019). Clinical Dementia Rating was administered for grading severity (0.5 = very mild; 1 = mild). Cerebrospinal fluid biomarkers were determined in selected cases according to the clinician’s criteria.

### Fluorodeoxyglucose Positron Emission Tomography Image Acquisition, Preprocessing, and Analysis

Fluorodeoxyglucose Positron Emission Tomography images were performed according to the guidelines of the European Association of Nuclear Medicine (Varrone et al., 2009). All images were obtained with the same scanner, a Siemens Biograph True Point PET/CT scanner. A mean dose of 185 MBq was administered 30 min before image acquisition and after at least 6 h of fasting. Images were acquired after sensory rest of patients. CT scan parameters were kVp/effective mAs/rotation: 130/40/1; slice thickness: 3 mm; reconstruction interval: 1.5 mm; and pitch: 0.75. PET images were acquired for 10 min at a single-bed position in sinogram mode. Images were reconstructed using an iterative reconstruction process (true X method with two iterations and 21 subsets).

Images were preprocessed and analyzed using Statistical Parametric Mapping software (SMP12), running in Matlab R2018A.^1^ Images were realigned and normalized to the standard Montreal Neurological Institute space using a brain FDG-PET template validated for dementia (Della Rosa et al., 2014). Global mean normalization was used for intensity scaling. A region of interest analysis was performed using *Marsbar* software, enabling the extraction of mean uptake values for each of the 116 brain regions of the Automatic Anatomical Labeling atlas for each patient.

In addition, a voxel-based brain mapping analysis was conducted with a *t*-test for two independent samples to compare each diagnostic group vs. 40 healthy controls. For these analyses, images were smoothed with a full width at half maximum of 12 mm, and age and sex were added to the statistical model as nuisance covariates. A family-wise error corrected *p* < 0.05 was used for multiple comparison correction, with an extent number of voxels of k = 30. These analyses were conducted to confirm the expected regions of hypometabolism according to each diagnostic group.

### Data Analysis

Our main aim was to design a framework tool based on artificial intelligence, particularly evolutionary machine Learning techniques, to perform automatic diagnosis of neurodegenerative diseases using FDG-PET images. However, the high dimensionality of data provided by PET image analysis requires derivation of techniques to reduce the dimensionality of the problem without impacting performance. Feature selection (also known as variable selection) can be defined as a combinatorial problem (NP-optimization problem) that aims to identify the smallest set of features (therefore, smallest set of input variables), or their combination, that maximizes a measure (Davies and Russe, 1994). In our case, FDG-PET imaging data using the Automatic Anatomical Labeling atlas included more than 100 features, which represents a high-dimensionality problem, as the number of variable combinations to be tested in the classification algorithm can be computed following the equation:


$$C=\sum_{r=1}^{n}\frac{n!}{r!\left(n-r\right)!}$$

In a previous study (Díaz-Álvarez et al., 2019), we addressed the problem of dimensionality reduction for automatic classification of PPA. In that study, we tested Principal Component Analysis (PCA) and four feature selection algorithms (*ChiSquaredAttributeEval*, *ClassifierAttributeEval*, *Cfs-SubsetEval*, and *WrapperSubsetEval*), and evaluated the performance of the classification process before and after the feature selection phase. PCA is a dimensionality reduction technique based on the orthogonal projection of the data on a more reduced linear space (principal component analysis). In contrast, feature selection techniques do not alter the representation of the variables, and select a subset of the original ones. Our research concluded that neither PCA nor any of the feature selection algorithms tested successfully improved the classification results. Moreover, the PCA only covered 88% of the variance, and the number of features remained high. As aforementioned, feature selection in high-dimensionality problems can hardly be accomplished by traditional procedures based on statistical techniques. Therefore, in this work we address the hypothesis that Evolutionary Algorithms can help to identify the best reduced set of relevant features and accomplish satisfactory automation of the diagnosis of neurodegenerative disorders using FDG-PET.

One of the most advanced evolutionary algorithms for feature selection is the Genetic Algorithm (GA). This is a stochastic method for function optimization based on the mechanics of natural genetics and biological evolution. In nature, the genes of organisms tend to evolve over successive generations to better adapt to the environment, and so does the GA to select the smallest set of input variables that maximizes the output measure. Moreover, GAs are one type of the metaheuristic algorithms (also known as population-based metaheuristic), that maintain and improve multiple candidate solutions using population characteristics to guide the search. One of the interesting capabilities of metaheuristics is their ability to extract themselves from a local minimum (Yang, 2014).

We designed a GA to assess the broad range of available features and to identify the set of the most relevant ones. The GA aimed to improve the automatic classification of patients with a diagnosis of AD or FTD. It was programmed in Matlab, and trained to obtain the best performance, in this case the highest fitness value (that is, the best candidate solution that maximizes the hit in diagnosis classification).

The general scheme of the GA designed is shown in Algorithm 1. The implementation of a GA requires the definition of the chromosome, that is the binary representation of solutions, and operators to apply on the chromosome. Supplementary Table 1 presents values for all the GA operators applied, that were obtained after a careful exploration of the space of solutions. These operators are:

**Table:** 

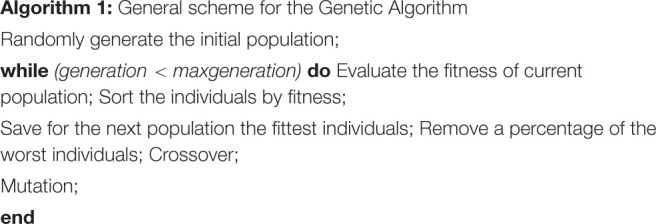

1. Generations: iteration steps of the GA.
2. Population size: number of candidate solutions that will be evolved.
3. Crossover: operator that allows more than one parent selected, and one or more offspring produced, using the genetic material of the parents. It is defined by its probability.
4. Mutation: operator that produces a small random tweak in the chromosome, to get a new solution.
5. Elitism: operator that involves copying a small proportion of the fittest candidates, unchanged, into the next generation. It is defined by its probability.

In this study, the number of generations and population size were fixed at 100 and 128, respectively. The individual’s chromosome has variable size, and every gene in the chromosome is an integer value, which represents a feature. The individual’s size and features were randomly selected for the initial population, from among all possibilities. The number of features depends on the dimensionality of the data contained in the working database.

A feature cannot be duplicated in an individual’s chromosome; if this occurs, a new feature will be randomly selected. Starting from the initial population, the evolutionary process is responsible for generating new individuals for the next population by applying the genetic operators (crossover, mutation, elitism). A percentage of the individuals with worst fit are eliminated in order to benefit the final result. Supplementary Figure 1 shows how the individual’s chromosome is built for the initial population. As a result, an individual is an *MxN* matrix, where *M* represents the number of instances and *N* the number of features (individual’s size). Features are randomly selected from the available features in the dataset to build the initial population. For each feature, the database is accessed in order to extract values assigned from the instances. If a feature is selected twice, one copy must be replaced.

New offsprings are generated by the crossover operator. Firstly, we used double tournament selection to randomly select two individuals from the population. Crossover is implemented as 1-point crossover; therefore, each chromosome is split into two sections. The first offspring contains the first chromosome section from parent *a*, and the second section from parent *b*. The second offspring contains the first chromosome section from parent *b*, and the second section from parent *a*. Mutation is performed by selecting a gene to be mutated, with a probability between 0 and 1, and replacing the feature with another selected randomly. In order to prevent duplication of features, a control mechanism was implemented and the feature replacement process is repeated until no duplication is achieved.

Supplementary Figure 2 shows the application of the selection and crossover operator. In this Figure, double tournament is used to select two members of the population and new offsprings are generated after applying the 1-point crossover operator.

The fitness function is defined as the execution of an unsupervised classification algorithm. Each individual, consisting of a variable number of features and their respective values for all instances, is provided as input to the classification algorithm. This algorithm returns the number of hits in the classification process, which is assigned to the individual as its fitness value. The maximum fitness is bounded by the total number of instances in the database. We selected two different classification algorithms to test our methodology: *BayesNet Naives* and *K-Nearest Neighbor*, which are integrated in the *Matlab Software environment* (the MathWorks Inc., 2018) and *R*2018*a* version, where our GA was implemented. These classification algorithms are available through *fitcnb* and *fitcknn*, respectively.

K-fold cross-validation was used for both classification algorithms. Cross-validation is one of the most widely used data resampling methods to assess the generalization ability of a predictive model and to prevent overfitting. To build the final model for the prediction of real future cases, the learning function (or learning algorithm) *f* is usually applied to the entire learning set. This final model cannot be cross-validated. The purpose of cross-validation in the model building phase is to provide an estimate for the performance of this final model on new data (Ranganathan et al., 2018).

K-fold cross-validation splits data into *k* subsets and performs *K* iterations to prevent overlapping. For each iteration, a different subset was chosen for testing, and the remainder for training. In this study, 5 and 3 values for *k* were considered appropriate to obtain an accurate estimation. *k* = 5 was selected for larger databases, and *k* = 3 for smaller datasets.

In the results section, we show the average results for 30 trials, for each dataset and set of features. Although we tested different numbers of generations and population sizes, the most relevant cutting was achieved with 100 and 128 as the number of generations and population size, respectively.

### Sample Size

The required training sample size for a particular machine learning model applied to clinical research data is often unknown. Characteristics of the sparsity of the sample, complexity of data, and employed methodology are conditioning factors of the sample size. This sample was considered appropriate for this study according to previous experience with similar data from a population that shares some clinical and neuroimaging characteristics (Matias-Guiu et al., 2018, 2019). Additionally, we followed a procedure of feature reduction that minimizes the required sample size and prevents overfitting. Finally, we performed a *post hoc* curve-fitting approach that requires empirical testing to model and extrapolate the algorithm performance as a function of sample size. Furthermore, K-fold validation was used to avoid overfitting and ensure the generalization capabilities of the achieved model.

### Data Availability

The conditions of our ethics approval do not permit public archiving of anonymized study data. Readers seeking access to the data should contact the corresponding author or the local ethics committee of Hospital Clinico San Carlos, Madrid. Access can be granted only to named individuals in accordance with ethical procedures governing the reuse of sensitive clinical data. The datasets used during the current study are available from the corresponding author after completion of a data sharing agreement and approval by the Ethics Committee.

## Results

### Voxel-Based Brain Mapping Results

**FIGURE 1:**
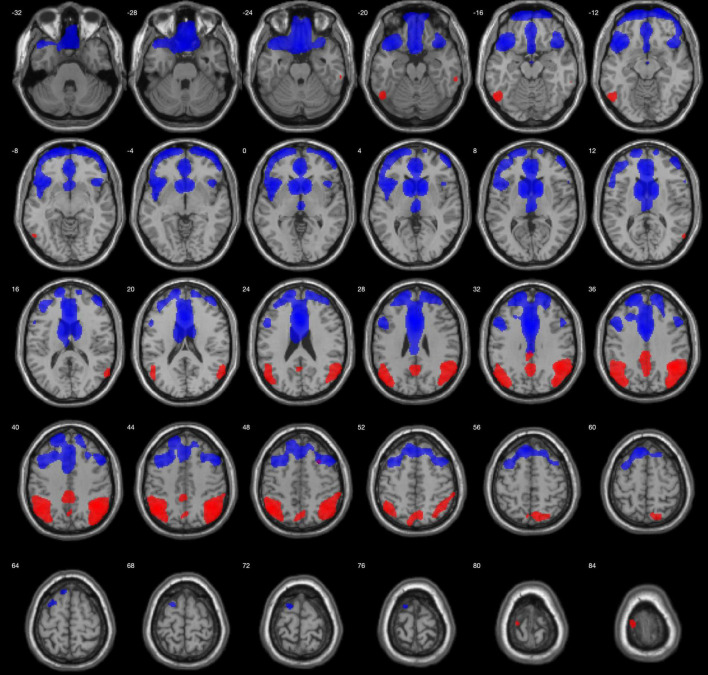
Regions with lower metabolism in the bvFTD group (blue) and AD (red) in comparison to HCs, displayed on MRI template. A 2-sample *t*-test with a family-wise error corrected *p* < 0.05 was used. Images are shown using neurological orientation.

**FIGURE 2:**
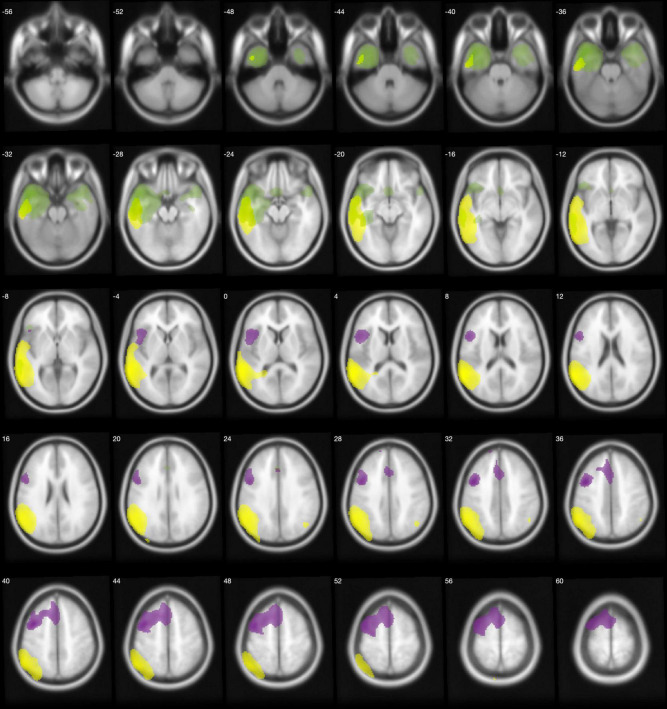
Regions with lower metabolism in the PPA variants displayed on an MRI template. nfPPA (violet), svPPA (green), and lvPPA (yellow) were compared with healthy controls using a 2-sample *t*-test with a family-wise error corrected *p* < 0.05. Images are shown using neurological orientation.

In comparison to the HC group, the AD group showed lower metabolism in both parieto-temporal lobes including the posterior cingulate and precuneus. Conversely, bvFTD showed lower metabolism mainly in both frontal lobes, as well as in the right anterior temporal lobe (Figure 1). Regarding PPA, nfPPA showed lower metabolism in the left frontal lobe; svPPA in bilateral anterior temporal lobe predominantly in the left side; and logopenic PPA was associated with left parieto-temporal hypometabolism (Figure 2).

### Discrimination Between Alzheimer’s Disease and Healthy Controls

**FIGURE 3:**
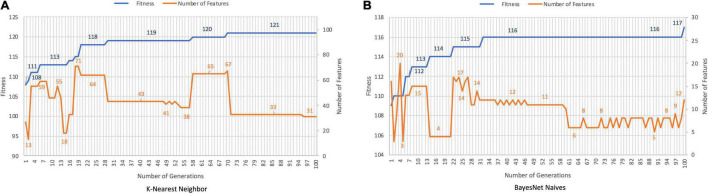
Results for AD records vs. HCs with *K-Nearest Neighbor* **(A)** and *BayesNet Naives* **(B)** as the fitness function. The ***X*** axis represents the generations, the main ***Y*** axis corresponds to the fitness value, and the secondary ***Y*** axis shows the number of features selected. The blue line represents the progression of fitness and the orange line the smallest set of features in the current generation.

The discrimination between patients with AD and HCs with the GA was achieved in 95.28% with *K-Nearest Neighbor* and 92.13% with *BayesNet Naives*. The number of features was decreased in 73.28% with *K-Nearest-Neighbor*, and 89.66% with *BayesNet Naives*. Figure 3 plots the fitness and number of features obtained by generation for *K-Nearest Neighbor* and *BayesNet Naives* as the fitness function. The number of features selected are 31 features for *K-Nearest Neighbor* and 12 for *BayesNet Naives* (Supplementary Table 2). The use of PCA to reduce the dimensionality required 66 features to cover the 88.3% of the variance.

### Discrimination Between Behavioral FTD and Healthy Controls

**FIGURE 4:**
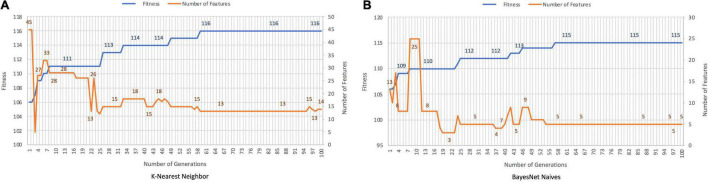
Results for bvFTD vs. HC with *K-Nearest Neighbor* **(A)** and *BayesNet Naives* **(B)** as the fitness function. The ***X*** axis represents the generation, the main ***Y*** axis corresponds to the fitness value, and the secondary ***Y*** axis shows the number of features selected. The blue line represents the progression of fitness and the orange line the smallest set of features in the current generation.

The GA discriminates between patients with bvFTD and HCs with an accuracy of 96.67 and 95.83%, with cutting rates of 87.93% for *K-Nearest Neighbor* and 95.69% for *BayesNet Naives*, respectively (Figure 4). Features selected are shown in Supplementary Table 2. Conversely, PCA required 59 features covering the 88.6 of variance.

### Differential Diagnosis Between Behavioral FTD and Alzheimer’s Disease

**FIGURE 5:**
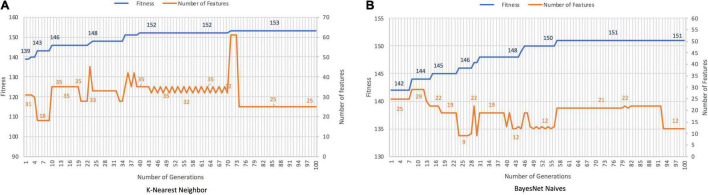
Results for bvFTD vs. AD, with *K-Nearest Neighbor* **(A)** and *BayesNet Naives* **(B)** as the fitness function. The ***X*** axis represents the generation, the main ***Y*** axis corresponds to the fitness value, and the secondary ***Y*** axis shows the features selected. The blue line represents the progression of fitness and the orange line the smallest set of features in the current generation.

*K-Nearest Neighbor* and *BayesNet Naives* achieved an accuracy of 90.53 and 89.35%, respectively for the discrimination between bvFTD and AD (Figure 5). The cutting rates were 78.45 and 89.66% for solutions with 25 and 12 features, respectively. In contrast, PCA needs 47 features covering the 88.8% of variance.

### Classification of Primary Progressive Aphasia Variants

**FIGURE 6:**
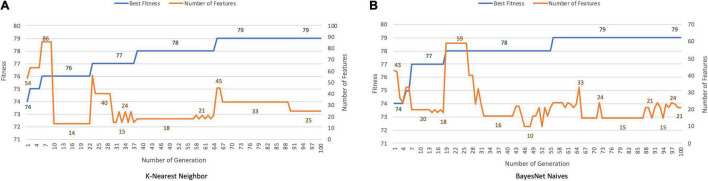
Results for PPA, with *K-Nearest Neighbor* **(A)** and *BayesNet Naive s* **(B)** as the fitness function. The ***X*** axis represents the generation, the main ***Y*** axis corresponds to the fitness value, and the secondary ***Y*** axis shows the features selected. The blue line represents the progression of fitness and the orange line the smallest set of features in the current generation.

Both classifiers, *K-Nearest Neighbor* and *BayesNet Naives*, found solutions with an accuracy rate of 90–91%. Figures 6A,B plot the results obtained with each classifier as the fitness function. The best solutions contained 25 and 21 of the initial 116 features, with cutting rates of 78.81 and 81.90%, respectively. When PCA was applied, 66 features were included, accounting for 88.5% of the variance.

### Performance Analysis of Best Classification Models

After the careful analysis of the previous results, *BayesNet Naives* algorithm showed the best performance in terms of fitness value for the four classification problems evaluated.

Then, we evaluated the performance of this algorithm for five quality metrics (accuracy, precision, sensitivity, F1-score, and specificity) in order to provide a deeper view of its classification capability. In particular, F1-score, as a measure of a test’s accuracy that considers both the precision and the recall of the test, gives a good insight of what to expect in terms of accurate binary decision.

Supplementary Table 3 shows the values of the quality metrics for the aforementioned classification problems. As can be seen, all the metrics achieve values very close to 1, and F1-score, as the compound metric, indicates an accurate discrimination between the two classes of the classification problem.

### Validation of the Alzheimer’s Disease Model With the Alzheimer’s Disease Neuroimaging Initiative Database

Although cross-validation techniques used along our model creation and validation assure the generalization of the approach, we have performed a further evaluation with data (FDG-PET images) obtained from the ADNI-3 database.^2^ The Alzheimer’s Disease Neuroimaging Initiative (ADNI) is an ongoing multi-site cohort study designed to characterize the trajectories of clinical, imaging, and fluid biomarkers across the entire spectrum of aging from clinically normal individuals through MCI to AD, with data made available publicly for widespread use (Weiner et al., 2013). For up-to-date information, see www.adni-info.org. ADNI-3 has several improvements, including the addition of tau and amyloid-PET (Weiner et al., 2017).

In this regard, we have created a new validation dataset composed of 22 PET images from AD patients, and 19 PET images from healthy controls. Supplementary Table 4 shows the main characteristics of the ADNI samples. These data have been provided to our AD vs. HC model as new data, and obtained the automatic classification of patients. The performance on the new data is measured with a set of quality metrics, including accuracy, precision, recall and F1 score, as shown in Supplementary Table 5. The last three give a better insight of the quality of the model and work well on balanced and unbalanced datasets. This model identified 86.36 and 95.45% of patients with AD diagnosis for *KNN* and *NB*, respectively. Both obtained high F1-scores.

## Discussion

Diagnosis and classification of patients with neurodegenerative disorders may be challenging. Because each neurodegenerative disorder has a relatively specific topographic pattern, patterns of cerebral glucose metabolism in FDG-PET imaging can be very useful for diagnosis. In this regard, different patterns of hypometabolism emerge when comparing each diagnostic group against HCs using whole-brain analyses. However, due to several factors such as individual variability or disease stage, these neuroimaging patterns are not entirely specific in clinical practice on an individual basis. In addition, some brain regions are more difficult to evaluate visually in early stages (Matias-Guiu et al., 2015a). Our study addresses the application of machine learning techniques to the diagnosis of AD, FTD, and related disorders.

Discrimination rate for AD vs. bvFTD, AD vs. HC, and bvFTD vs. HCs was very high, 90.53, 95.28, and 96.67%, respectively. These values represent a better classification than previously reported works (Nestor et al., 2018). For instance, Rabinovici et al. (2011) showed a sensitivity of 77.5% and a specificity of 98% for visual analysis in a group of 62 patients with AD and 45 patients with FTD (behavioral variant or aphasic variants). Our approach obtained a sensitivity of 97.7, 96.6, and 96.3%, and a specificity of 100, 92.3, and 84.6%. Comparing with the validation process with the ADNI dataset, our results showed a sensitivity of 86.36 and 95.45%, although they present lower specificity values (68% for NB). In addition, our classification rate was also higher than achieved by applying other machine learning algorithms to cortical thickness (Kim et al., 2019).

In this scenario, genetic algorithms can be designed efficiently to cope with a large set of input features, explore a wide range of solutions, and avoid local minima in a way that is difficult to achieve with traditional machine learning techniques. Regarding PPA, classification accuracy was also high but was incomplete. This may be explained by a certain overlap between PPA variants, which hinders the diagnosis and constitutes a controversial issue in the field of PPA (Sajjadi et al., 2012). In this regard, we recently suggested that PPA may be categorized into five variants based on brain metabolism (Matias-Guiu et al., 2018). Furthermore, one of our most striking results was the GA’s ability to reduce the number of features. This was especially valuable for differentiation between patients with AD and HCs, patients with bvFTD and HCs, and between AD and bvFTD, in which a limited set of 5–12 features achieved good accuracy. Regions selected by the GA included several gyri previously linked to these disorders in early stages, such as the precuneus and some parietal and temporal regions in AD, and the anterior cingulate, and several frontal gyri in bvFTD. In contrast, the cutting rate was lower for differential diagnosis of PPA variants, and a higher number of features was necessary. Several features involving mainly frontal, temporal, and parietal lobes of the left and right hemispheres were included in the GA. This may be explained by the regional overlap between PPA variants, which explains the clinical and neuroimaging similarities between PPA subtypes. Since our methodology addressed a feature selection approach applying a GA to FDG-PET data images, we launched 30 trials in order to reduce the influence of chance. Table 2 shows the average values for each trial. On average, the best solutions reached accuracy rates generally between 85 and 92% for most of the experimental tests. Regarding the number of features, the average cutting rate was always higher than 51% and higher than 64% for most of the results. These data suggest that, even when considering average results, GA were able to find solutions that improve the accuracy with a features cutting rate higher than 50%. The results achieved by the genetic algorithm are highly generalizable as the tuning of the parameters of the algorithm and training phase were carefully selected to avoid overfitting, and the sample used for the training is large enough and representative of the potential use in clinical practice.

**TABLE 2:** Average results for FDG-PET imaging data.

**Fitness function**	**Fitness**	**Fitness rate (%)**	**Features**	**Cutting rate (%)**
**AD vs. HCs**				
*K-Nearest Neighbor*	115.11	90.64	47.96	58.66
*BayesNet Naives*	113.76	89.58	18.38	84.15
**bvFTD vs. HCs**				
*K-Nearest Neighbor*	111.09	92.57	31.93	72.48
*BayesNet Naives*	110.84	92.36	17.19	85.18
**bvFTD vs. AD**				
*K-Nearest Neighbor*	145.30	85.98	39.96	65.55
*BayesNet Naives*	145.34	86.12	28.60	75.34
**PPA variants vs. HCs**				
*K-Nearest Neighbor*	76.23	89.69	56	51.77
*BayesNet Naives*	76.83	90.39	41	64.48

*Decimal numbers represent average values.*

Logistic regression analysis of FDG-PET results with some clinical or genetic data has been used to predict the chance of conversion to dementia in patients with mild cognitive impairment (Arbizu et al., 2013). Machine learning constitutes a powerful approach for the automation of neuroimaging-based diagnosis of neurodegenerative disorders. Due to the heterogeneity of these disorders, automation and feature selection is challenging. However, traditional machine learning approaches have failed to achieve highly accurate results in complex diagnostic problems (Davatzikos, 2019) like the one presented in this study. Characteristics such as a large set of input features, and the lack of a large database with thousands of instances per target class, encourage the development of novel approaches. Genetic algorithms are a new computational technique based on an analogy with Darwin’s theory of natural selection. They include optimization methods based on iterative search, enabling the evaluation of several solutions or hypotheses in parallel, and even their recombination. Moreover, genetic algorithms operate on a population of individuals to produce increasingly accurate approximations, and do not require *a priori* knowledge of the problem under study. To our knowledge, this strategy has not previously been applied to PET imaging in dementia. Interestingly, our results show several combinations of features that achieved high diagnostic capacity using only FDG-PET imaging. The reduced number of features selected with the algorithms suggests that diagnosis may be easily automated in future studies.

Another interesting approach is the combination of data from several diagnostic techniques. In this regard, Gupta et al. (2019) proposed a novel method for extracting data from PET and structural MRI and then combining these features with CSF and APOE genotype for the discrimination between AD, Mild Cognitive impairment, and healthy controls. In their study, the authors used truncated singular value decomposition (TSVD), which is another approach to reduce dimensionality. This method is similar to PCA. However, factorization is performed on the data matrix, whereas in PCA is conducted on the covariance matrix. Our approach is mainly based on the application of genetic algorithms to conduct the feature selection process. The genetic algorithm is a stochastic method for function optimization that explores a much broader space of solutions, as there is no limit for how many features the algorithm can choose. Besides, traditional methods of feature selection essentially entail trying out all the combinations of potential features. Genetic feature selection takes a different approach—it learns from an exploration/exploitation trade-off, searching a larger search space and arriving at a better solution in less time.

Our study has some limitations. First, diagnosis was not pathologically confirmed. However, all patients were comprehensively examined, met the current diagnostic criteria, and were followed-up for at least 2 years at a center with extensive experience in these disorders. Second, all cases were evaluated at the same center with the same PET scanner. However, we performed a cross-validation for all the algorithms, and an external validation using independent data from ADNI was performed in the case of AD vs. healthy controls. Third, we only included FDG-PET imaging in the analyses. Future studies including other diagnostic techniques (other PET tracers, MRI, etc.) may be interesting for head-to-head comparisons.

In conclusion, our study supports the use of FDG-PET imaging in the diagnosis of AD and FTD. The application of genetic algorithms to FDG-PET identified the most relevant brain regions, which may be useful as features for the automated diagnosis of neurodegenerative disorders. According to our results, the use of such metaheuristic techniques as genetic algorithms is probably an optimal strategy for identifying the most relevant features and maximizing diagnostic accuracy. Overall, our study contributes to progress toward automated and optimized diagnosis of neurodegenerative disorders using FDG-PET imaging.

## Data Availability Statement

The raw data supporting the conclusions of this article will be made available by the authors, without undue reservation.

## Ethics Statement

The studies involving human participants were reviewed and approved by the Comité de Ética e Investigación Clínica (CEIC) Hospital Clínico San Carlos. The patients/participants provided their written informed consent to participate in this study.

## Author Contributions

JA and JAM-G: conceptualization. MC-M, VP, and JAM-G: data acquisition. JD-Á, JAM-G, MC-M, FG-G, IS-R, and JA: methodology. JD-Á, JA, and JAM-G: writing original draft preparation. FG-G, JD-Á, LH-L, and JA: formal analysis and investigation. JM-G, JAM-G, JD-Á, and JA: funding acquisition. JD-Á, JM-G, JA, JAM-G, and JC: supervision. JD-Á, JAM-G, MC-M, VP, IS-R, FG-G, LH-L, JM-G, JC, and JA: writing review and editing. All authors contributed to the article and approved the submitted version.

## Conflict of Interest

The authors declare that the research was conducted in the absence of any commercial or financial relationships that could be construed as a potential conflict of interest.

## Publisher’s Note

All claims expressed in this article are solely those of the authors and do not necessarily represent those of their affiliated organizations, or those of the publisher, the editors and the reviewers. Any product that may be evaluated in this article, or claim that may be made by its manufacturer, is not guaranteed or endorsed by the publisher.

## Footnotes

1
https://www.fil.ion.ucl.ac.uk/spm-statistical-parametric-mapping/

2
adni.loni.usc.edu
