# Supplementary material for: Genetic Algorithms for Optimized Diagnosis of Alzheimer’s Disease and Frontotemporal Dementia Using Fluorodeoxyglucose Positron Emission Tomography Imaging
Source: Front Aging Neurosci. 2022 Feb 3;13:708932. doi: 10.3389/fnagi.2021.708932 (PMC8851241; doi:10.3389/fnagi.2021.708932)
Supplement: Supplementary file 1 [file Table_1.DOCX]

| **Supplementary Table 1. Genetic operators** | |
| --- | --- |
| **Operator** | **Value** |
| Generations | 100 |
| Population size | 128 |
| Crossover | 0.6 |
| Mutation | 0.01 |
| Elitism | 0.3 |
