# Supplementary material for: Genetic Algorithms for Optimized Diagnosis of Alzheimer’s Disease and Frontotemporal Dementia Using Fluorodeoxyglucose Positron Emission Tomography Imaging
Source: Front Aging Neurosci. 2022 Feb 3;13:708932. doi: 10.3389/fnagi.2021.708932 (PMC8851241; doi:10.3389/fnagi.2021.708932)
Supplement: Supplementary file 2 [file Table_2.DOCX]

Supplementary Table 2: Selected features for the best solutions for differential diagnosis of AD and bvFTD, and PPA, with classifiers *BayesNet Naives (NB)* and *K-Nearest-Neighbor (KNN)*. Features are presented in alphabetical order.

| **Classifier** | **Features** |
| --- | --- |
| **AD vs HC** | |

| KNN | Angular L, Angular R, Calcarine R, Cerebellum 10 R, Cerebellum 3 L, Cerebellum 4 5 R, Cerebellum 6 L,  Cerebellum 7b L, Cerebellum 8 L, Cerebellum 8 R, CingulumPost R, Frontal Inf Oper R,  Frontal Inf Orb L, Frontal Inf Tri L, Frontal Med Orb R, Frontal Mid Orb L, Frontal Sup Orb R, Heschl R, Hippocampus L, Precentral R, Precuneus L, Precuneus R, Putamen R, Rectus R, TemporalInf L, TemporalPole Mid R, TemporalPole Sup R, TemporalSup R, Thalamus L, Thalamus R, Vermis 4 5 |
| --- | --- |
| NB | Angular L, Calcarine L, Calcarine R, Cerebellum 10 R, Cerebellum 8 R, CingulumMid R, Cuneus L,  Occipital Inf R, Olfactory L, ParietalSup L, TemporalSup L, Thalamus R |

**bvFTD vs HC**

| KNN | Cerebellum 9 R, CingulumAnt L, CingulumMid R, Frontal Inf Oper R, Frontal Sup Orb L, Occipital Mid L,  Pallidum R, ParietalSup L, Precentral L, Rectus R, TemporalPole Mid L, Vermis 6, Vermis 8 |
| --- | --- |
| NB | CingulumAnt L, Frontal Inf Oper R, Occipital Mid L, ParietalSup L, TemporalSup L |

**bvFTD vs AD**

| KNN | Amygdala R, Caudate L, Cerebellum 10 L, Cerebellum 3 R, Cerebellum 7b L, Cerebellum Crus1 L,  Cerebellum Crus1 R, CingulumPost R, Cuneus R, Frontal Inf Oper L, Frontal Med Orb L, Frontal Mid L, Frontal Mid Orb R, Frontal Sup Orb L, Frontal Sup Orb R, Fusiform R, Occipital Mid R, Occipital Sup R, Olfactory R, Pallidum L, ParietalInf L, Rolandic Oper L, TemporalPole Mid R, TemporalPole Sup R, Thalamus L |
| --- | --- |
| NB | Frontal Inf Oper L, Frontal Inf Orb L, Frontal Inf Orb R, Occipital Inf L, Occipital Sup L, ParietalInf R,  ParietalSup R, Putamen R, TemporalInf R. TemporalPole Mid L, TemporalSup L, Thalamus R |

**PPA (differential diagnosis between different variants and HCs**

| KNN | Amygdala L, Calcarine R, Cerebellum 8 L, Cerebellum 8 R, Frontal Inf Oper L, Frontal Inf Oper R,  Frontal Inf Orb L, Frontal Inf Orb R, Frontal Mid L, Frontal Mid Orb R, Frontal Mid R, Frontal Sup L, Frontal Sup R, Fusiform L, Fusiform R, Lingual R, Occipital Inf R, Occipital Mid R, Pallidum L, SMA L, SMA R, TemporalMid R, TemporalPole Mid R, TemporalPole Sup L, Vermis 10 |
| --- | --- |
| NB | Calcarine L, Caudate R, Cerebellum Crus2 L, Cuneus R, Frontal Inf Tri L, Heschl R, Insula L,  Occipital Inf R, Parahippocamp L, Parahippocamp R,Precentral L, Putamen L, Rectus R, SMA R, Supramarginal R, TemporalMid R, TemporalPole Mid R, TemporalPole Sup L, Thalamus L, Vermis 1 2, Vermis 8 |
