# Supplementary material for: Genetic Algorithms for Optimized Diagnosis of Alzheimer’s Disease and Frontotemporal Dementia Using Fluorodeoxyglucose Positron Emission Tomography Imaging
Source: Front Aging Neurosci. 2022 Feb 3;13:708932. doi: 10.3389/fnagi.2021.708932 (PMC8851241; doi:10.3389/fnagi.2021.708932)
Supplement: Supplementary file 3 [file Table_3.DOCX]

Supplementary Table 3: Performance of datasets with the best solution obtained by *BayesNet Naives (NB)*.

| **Experiment** | **Accuracy** | **Precision** | **Sensitivity** | **F1-score** | **Specificity** |
| --- | --- | --- | --- | --- | --- |
| *AD vs. HC* | 0.9528 | 0.9659 | 0.9659 | 0.9659 | 0.9231 |
| *FTD vs. HC* | 0.9925 | 0.9286 | 0.9630 | 0.9455 | 0.8462 |
| *AD vs. FTD* | 0.9882 | 1 | 0.9773 | 0.9885 | 1 |
| *PPA variants* | 0.8676 | 0.95 | 0.8261 | 0.8837 | 0.9778 |
