# Supplementary material for: Genetic Algorithms for Optimized Diagnosis of Alzheimer’s Disease and Frontotemporal Dementia Using Fluorodeoxyglucose Positron Emission Tomography Imaging
Source: Front Aging Neurosci. 2022 Feb 3;13:708932. doi: 10.3389/fnagi.2021.708932 (PMC8851241; doi:10.3389/fnagi.2021.708932)
Supplement: Supplementary file 4 [file Table_4.DOCX]

| **Supplementary Table 4**. Main demographic characteristics of the validation cohort of AD and HC from ADNI-3 | | |
| --- | --- | --- |
|  | AD (n=22) | HC (n=19) |
| Age (years) | 73.65±9.87 | 76.48±4.09 |
| Women n (%) | 7 (32%) | 11 (58%) |
| Years of education | 15.73±2.60 | 16.95±1.99 |
| MMSE | 21.91±3.45 | 29.26±1.15 |
