# Supplementary material for: Genetic Algorithms for Optimized Diagnosis of Alzheimer’s Disease and Frontotemporal Dementia Using Fluorodeoxyglucose Positron Emission Tomography Imaging
Source: Front Aging Neurosci. 2022 Feb 3;13:708932. doi: 10.3389/fnagi.2021.708932 (PMC8851241; doi:10.3389/fnagi.2021.708932)
Supplement: Supplementary file 5 [file Table_5.docx]

Supplementary Table 5: Performance in the ADNI validation dataset with classifiers *K-Nearest-Neighbor (KNN)* and *BayesNet Naives (NB)*.

| **Classifier** | **Accuracy** | **Precision** | **Sensivity** | **F1-score** |
| --- | --- | --- | --- | --- |
| *KNN* | 0.6585 | 0.6333 | 0.8636 | 0.7308 |
| *NB* | 0.8293 | 0.7778 | 0.9545 | 0.8571 |
