# Supplementary material for: Genetic Algorithms for Optimized Diagnosis of Alzheimer’s Disease and Frontotemporal Dementia Using Fluorodeoxyglucose Positron Emission Tomography Imaging
Source: Front Aging Neurosci. 2022 Feb 3;13:708932. doi: 10.3389/fnagi.2021.708932 (PMC8851241; doi:10.3389/fnagi.2021.708932)
Supplement: Supplementary file 6 [file Data_Sheet_1.PDF]

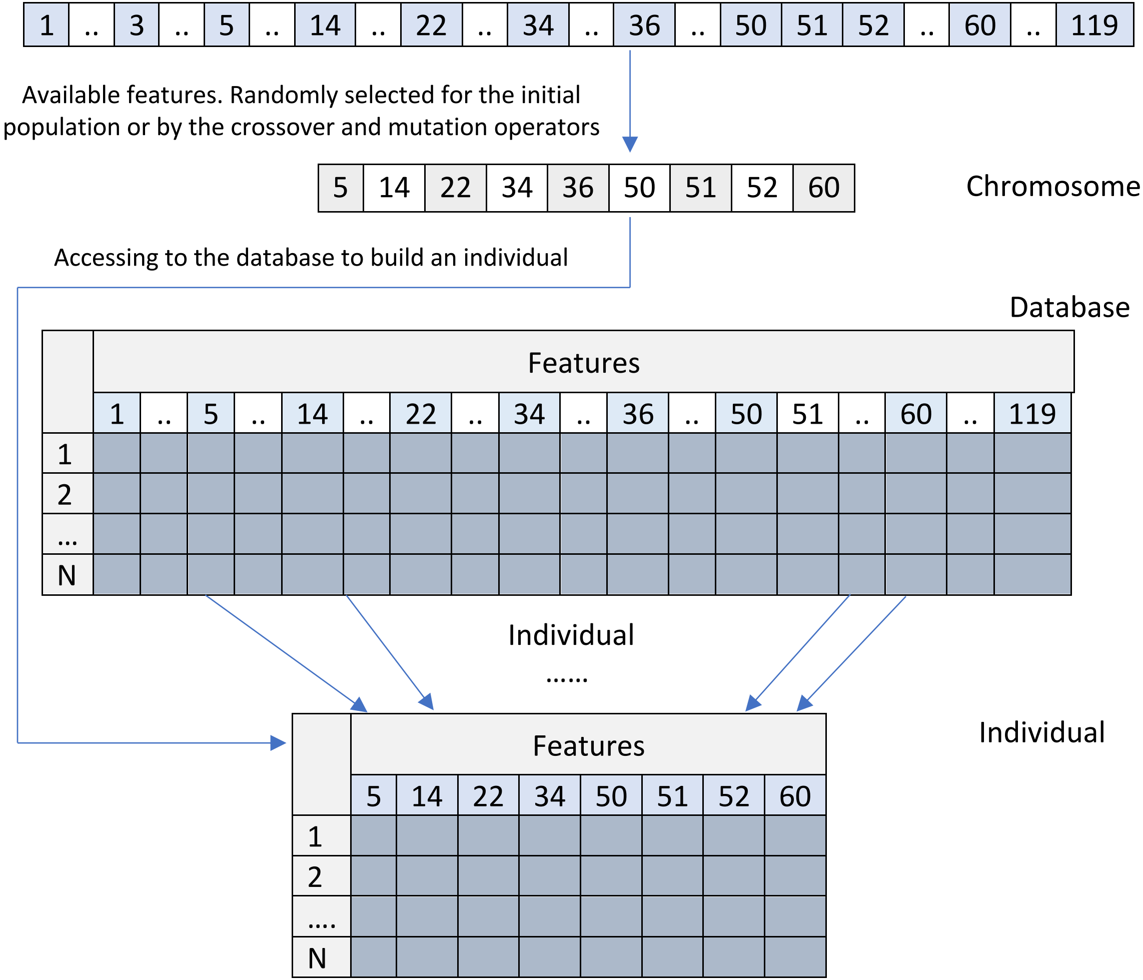
1 .. 3 .. 5 .. 14 .. 22 .. 34 .. 36 .. 50 51 52 .. 60 .. 119
Available features. Randomly selected for the initial population or by the crossover and mutation operators
5 14 22 34 36 50 51 52 60
Chromosome
Accessing to the database to build an individual
Database
Features
1 .. 5 .. 14 .. 22 .. 34 .. 36 .. 50 51 .. 60 .. 119
1
2
...
N
Individual
......
Individual
Features
5 14 22 34 50 51 52 60
1
2
....
N
