# Supplementary figures and images for: Genetic Algorithms for Optimized Diagnosis of Alzheimer’s Disease and Frontotemporal Dementia Using Fluorodeoxyglucose Positron Emission Tomography Imaging
Source: Front Aging Neurosci. 2022 Feb 3;13:708932. doi: 10.3389/fnagi.2021.708932 (PMC8851241; doi:10.3389/fnagi.2021.708932)

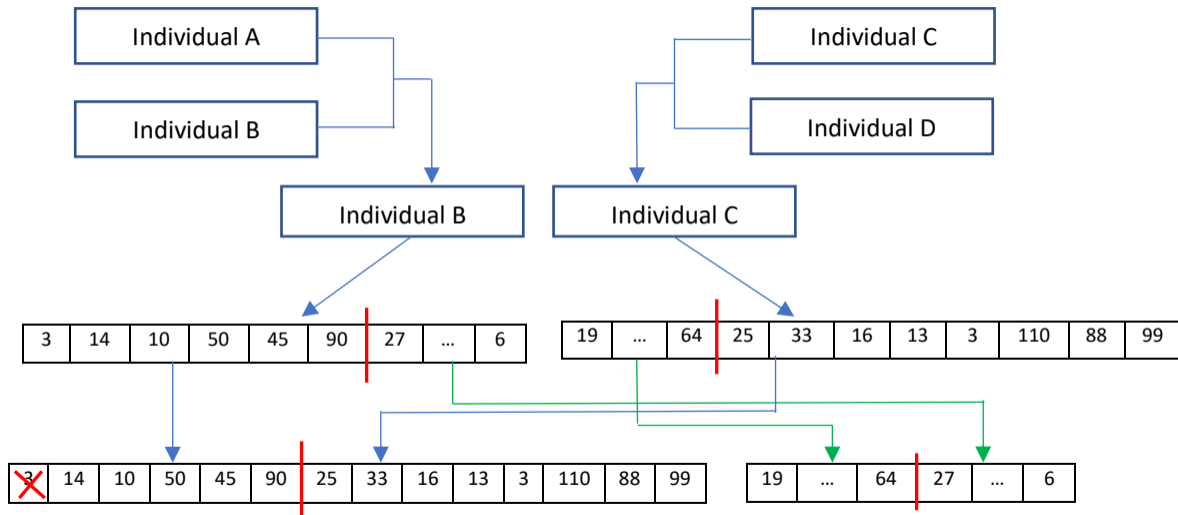

Supplement: Supplementary file 7 [file Data_Sheet_2.PDF]
